# Proteomics of Galápagos Marine Iguanas Links Function of Femoral Gland Proteins to the Immune System

**DOI:** 10.1074/mcp.RA120.001947

**Published:** 2020-11-25

**Authors:** Frederik Tellkamp, Franziska Lang, Alejandro Ibáñez, Lena Abraham, Galo Quezada, Stefan Günther, Mario Looso, Fabian Jannik Tann, Daniela Müller, Franz Cemic, Jürgen Hemberger, Sebastian Steinfartz, Marcus Krüger

**Affiliations:** 1 CECAD Research Center Institute for Genetics, University of Cologne, Cologne, Germany; 2 TRON - Translational Oncology at the University Medical Center of the Johannes Gutenberg University Mainz, Mainz, Germany; 3 Department of Comparative Anatomy, Institute of Zoology and Biomedical Research, Jagiellonian University, Kraków, Poland; 4 Dirección Parque Nacional Galápagos, Puerto Ayora, Santa Cruz, Galápagos, Ecuador; 5 Max Planck Institute for Heart and Lung Research, Bad Nauheim, Germany; 6 Institute for Biochemical Engineering and Analytics (IBVA), Giessen, Germany; 7 Molecular Evolution and Systematics of Animals, University of Leipzig, Leipzig, Germany; 8 Center for Molecular Medicine Cologne (CMMC), University of Cologne, Cologne, Germany

**Keywords:** Proteomics, marine iguana, femoral glands, immune system, protease inhibitor protein identification, database design, animal models, evolution, tissues, protease inhibitor

## Abstract

Communication between individuals via molecules, termed chemosignaling, is widespread among animal and plant species. However, we lack knowledge on the specific functions of the substances involved for most systems. The femoral gland is an organ that secretes a waxy substance involved in chemical communication in lizards. Although the lipids and volatile substances secreted by the femoral glands have been investigated in several biochemical studies, the protein composition and functions of secretions remain completely unknown. Applying a proteomic approach, we provide the first attempt to comprehensively characterize the protein composition of femoral gland secretions from the Galápagos marine iguana. Using samples from several organs, the marine iguana proteome was assembled by next-generation sequencing and MS, resulting in 7513 proteins. Of these, 4305 proteins were present in the femoral gland, including keratins, small serum proteins, and fatty acid-binding proteins. Surprisingly, no proteins with discernible roles in partner recognition or inter-species communication could be identified. However, we did find several proteins with direct associations to the innate immune system, including lysozyme C, antileukoproteinase (ALP), pulmonary surfactant protein (SFTPD), and galectin (LGALS1) suggesting that the femoral glands function as an important barrier to infection. Furthermore, we report several novel anti-microbial peptides from the femoral glands that show similar action against *Escherichia coli* and *Bacillus subtilis* such as oncocin, a peptide known for its effectiveness against Gram-negative pathogens. This proteomics data set is a valuable resource for future functional protein analysis and demonstrates that femoral gland secretions also perform functions of the innate immune system.

## Teaser

Femoral glands secrete a wax-like substance on the inner side of lizard hind legs, which is thought to function as a mode of chemical communication. Though the minor volatile fraction is well studied, the major protein fraction remains enigmatic. Here, we use proteomics to analyze proteins in femoral gland secretions of the Galápagos marine iguana. Although we found no evidence for proteins and peptides involved in chemical communication, we found several immune-regulatory proteins which also demonstrate anti-microbial functions. Accordingly, we show that femoral gland proteins and peptides function as a barrier against microbial infection and may prevent the rapid degradation of volatile substances.

## Graphical Abstract

**Figure:**
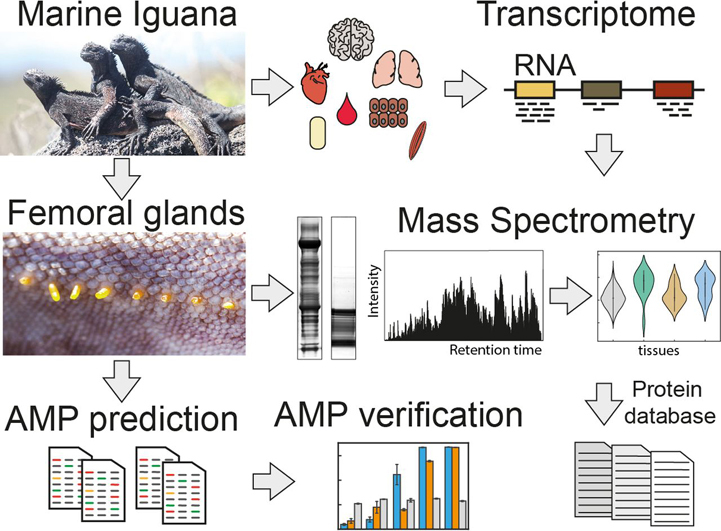

**Highlights**

• Comprehensive transcriptome and proteome of the Galápagos Marine Iguana.
• Analysis of femoral gland proteome.
• Identification of antimicrobial activity in femoral gland secretions.

Femoral glands are small epidermal skin glands located on the lateral inner side of the hind legs of many lizards and amphibians and play a central role in the chemical signaling systems of nonophidian squamates (1, 2). Additionally femoral gland secretions are used as chemosignals for communication, territory marking, signaling of male quality, as well as self-, sex- and species recognition (3, 4). Femoral glands are holocrine glands and release a waxy plug through their pores, which is composed of secreted proteins and also terminally differentiated glandular cells. As the secretory plug emerges from the body surface, mechanical forces such as friction on rock surfaces disperse the secreted material and release cellular compounds and volatile substances. Gland secretions are a mixture of proteins (∼80%), lipids (∼20%), and volatile molecules. Although the chemical composition and chemosignaling functions of the lipid fraction have been analyzed in a range of biological contexts (5, 6), the identity and functions of the proteins in femoral gland secretions remain completely unknown (7).

Marine iguanas (*Amblyrhynchus cristatus*) are endemic to the Galápagos islands and are one of the few squamate species in which single males defend a resource-less territory to attract females during the reproductive season. This lek system can be found in several species including birds, fish, reptiles, and mammals (8) but is rare in lizards.

Marine iguanas exhibit territorial behavior and sexual dimorphic coloration. Males attract females via specific lipophilic substances released by their femoral glands. Certain lipids of femoral gland secretions of male marine iguanas are thought to be involved in territory marking and for communication of male quality to females (9).

Mass spectrometry-based proteomics is a useful approach to investigate protein expression in tissues and body fluids. However, proteomics requires established databases containing annotations from a reference genome, which were not previously available for the marine iguana. Until now, the phylogeny and population structure of marine iguanas have mainly been studied in an evolutionary context by analysis of mitochondrial genes and standard nuclear genes, or by detection of genome-wide polymorphisms based on restriction site-associated DNA (RAD) sequencing (10). The UniProtKB counts only 75 protein entries (73 of them unreviewed) for marine iguanas, illustrating our rudimentary knowledge of protein annotations for this unique iguanid species.

By assembling the first proteomic data set for marine iguanas, we sought to identify and determine the biological functions of the proteins present in femoral gland secretions of lizards. Next-generation sequencing of multiple tissues and *de novo* assembly of the transcriptome have been successfully applied in several other organisms with unknown genomes, including the newt (*Notophthalmus viridescens*) (11) and the African clawed frog (*Xenopus laevis*) (12). By sequencing the transcriptome of various tissues, we were able to establish a comprehensive protein database and identify over 7,500 proteins in the marine iguana. Furthermore, sequence homology searches enabled us to correlate proteins secreted by the femoral gland with functions relating to lipid transport, protease inhibition, and activation of the immune system.

## EXPERIMENTAL PROCEDURES

See SI Methods for complete details.

##### Tissue Harvesting

Several tissues and secretions (brain, erythrocytes, femoral gland secretions, heart, lung, muscle, serum, sperm, and skin tissue) were harvested from an individual marine iguana from Genovesa island that had died of natural causes. The Galápagos National Park authority granted the research permission for this study.

##### Transcriptomics

Sequencing was performed on a NextSeq500 instrument (Illumina, San Diego, California) using v2 chemistry, resulting in an average of 80 to 240 M reads per library with the 2 × 150 bp paired end setup. The resulting raw reads were assessed for quality, adapter content, and duplication rates using FastQC (13). Trimmomatic version 0.33 was employed to trim reads if quality dropped below a mean of Q18 in a window of five nucleotides (14). Only reads above 30 nucleotides were selected for further analyses.

##### Assembly of ORFs

For *de novo* assembly of mRNA reads, Trinity (v2.0.6) was used in RF-stranded mode (–SS_lib_type RF) with minimum contig criteria (–min_contig_length 300 –min_kmer_cov 2) (15). ORF detection of assembled contigs was performed with the transcoder package (http://transdecoder.github.io) using the TransDecoder.LongOrfs command with option -S for strand specific assemblies, only analyzing the top strand. Duplicate ORFs were removed and interlaced ORFs were melted using the CD-HIT package (http://weizhongli-lab.org/cd-hit/) employing the parameters (-c, 1.00 and-n, 5).

##### Protein Isolation, Digestion and LC–MS/MS Analysis

After in-gel digestion with trypsin and peptide desalting, reverse phase liquid chromatography was used to separate peptides using an Easy nLC 1000 UHLPC. A hybrid quadrupole Orbitrap instrument (QExactive, Thermo Fisher Scientific, Waltham, Massachusetts) was used for mass spectrometric analysis (16). Each gel fraction was measured with a 150 min LC–MS/MS gradient.

##### MS Data Processing

Raw MS data files were analyzed using MaxQuant software (1.5.3.8) (17). We used the translated ORFs from the marine iguana as protein database, combined with known common contaminations. Default settings were used and peptides and proteins were identified using a false discovery rate (FDR) of 1% with intensity based absolute quantification (iBAQ) being enabled. Minimum peptide length was 7 amino acids. We allowed single peptide identifications and identifications by degenerated peptides, termed razor peptides, in this manuscript and defined this way proteins groups. Proteins or protein groups identified by a single peptide are only annotated as such in Additional file 1.

##### Protein Annotation

We performed protein BLAST searches against the UniProt and SwissProt databases using the Bio.Blast module of Biopython (18). We searched against 11 organisms, including the American alligator, Chinese alligator, green iguana, land iguana, marine iguana, Carolina anole, chicken, human, mouse, and rat, with an E-value cutoff of E-20. BLAST-hits with the lowest E-value were extracted for every organism and used for annotation. Protein sequences without similarities were searched against the UniProt and SwissProt *E. coli* databases to exclude bacterial contaminants.

##### Experimental Design and Statistical Rationale

One dead animal was used for the initial assessment of the samples and statistical analysis was not applicable. Data analysis and visualization were applied as follows:

Data analysis was performed using Perseus (19), the R environment (20), and Instant Clue (21) (http://www.instantclue.uni-koeln.de/). Human gene ontology terms for marine iguana proteins were based on the closest UniProt hits in BLAST searches. To identify proteins enriched in femoral gland secretions in an unbiased manner, we employed a strategy combining kmeans clustering and principal component analysis ([PCA]; see SI Appendix, SI Materials and Methods). We identified proteins with a valid intensity value in femoral gland secretions and at least two more values in any other tissue. Because we identified a small number of proteins in serum, we did not include this sample in our later analysis.

To further characterize the remaining proteins for which homologs were not detected, we performed database searches using several online tools. DeepLoc was used to predict subcellular localization using neuronal networks (22) and signal peptide cleavage sites were predicted using SignalP (23). To provide functional annotations for proteins without any sequence-related proteins in other organisms, we used HMMER to search against the Pfam protein profile/domain database (24). In addition a BLAST search was performed against UniProt and SwissProt protein databases without any E-value cutoff. Resulting hits were sorted according to their E-value and grouped into three confidence classes: (1) E-values <1E-20; (2) E-values 1E-20 – 1E-05; (3) E-values >1E-05.

##### In Silico AMP Prediction

Four support vector machines (SVM) with rbf (radial basis function, gaussian), linear, polynomial and sigmoid kernels were trained using Pythons scikit-learn (25) for antimicrobial peptide sequences (AMPs). Known AMP sequences were retrieved from the CAMP (Collection of Anti-Microbial Peptides) database (26) and APD (Antimicrobial Peptide Database) (27). Sequences had to fulfill the following requirements: manually curated and experimentally validated CAMP sequences, length of 5–100 amino acids and sequence identity <90% to each other.

Physicochemical features, including Chou's pseudo amino acid composition (28, 29) were calculated with Biopython (18) and Propy (30). Feature selection was performed with a principle component analysis in R. The SVMs were trained with an exhaustive grid-search and 3-fold cross-validation (CV). The accuracy of the best kernels given by sklearn and the corresponding area under the curve (AUC) of the receiver operation characteristic are shown in (Additional file 2). The list of predicted AMPs was further inspected manually and only peptides with a predicted high water solubility and chemical stability were chosen for the synthesis.

##### Antimicrobial Activity

Test strains of Bacillus subtilis, Escherichia coli K12 or Escherichia coli BL21 were grown in Miller-Hinton broth (MHB) for 18–20 h at 37 °C with moderate shaking. An inoculum of 1–2 × 107 cfu/ml was prepared in fresh MHB and 100 µl of the suspension was pipetted into microtiter plate wells together with 20 µl test substance. Test substances were measured in duplicates. The plates were covered by a plastic seal to prevent evaporation and incubated for further 18–24 h at 37 °C with moderate shaking. The test substances were dissolved in water at a concentration of 1 mg/ml and a 2n dilution series down to 31 µg/ml was prepared in sterile PBS. After incubation, the bacterial growth was estimated with a Photometer at 600 nm and the relative growth inhibition was calculated compared with a control without test substance. As positive control the well-studied AMP oncocin was used.

##### Peptide Synthesis

Peptide synthesis was performed using an automatic, microwave-based solid phase peptide synthesizer using the FMOC method at 0.1 mMol scale (31). DIC/Oxyma served as activation solution, coupling was performed at 75 °C, deprotection was performed with 20% piperidine in DMF at 90 °C. After synthesis the beads were washed with DMF and lyophilized. Cleavage of the product from the resin was performed with 95% TFA, 2.5% H_2_O, 2.5% TRIS for 4 h at room temperature. The resin was removed by filtration and the product precipitated with ether at −80 °C, centrifuged and washed twice with the same solvent. After removal of the ether, the peptide was dissolved in water. The peptides were purified by RP-HPLC on a C18/100 Å column using a water/acetonitrile gradient in the presence of 0.08% TFA. Identity of the peptides was verified by MALDI-TOF-MS. Peptides were stored frozen at −80 °C.

## RESULTS

##### Database Generation and Proteomic Analysis of the Marine Iguana

**Fig. 1:**
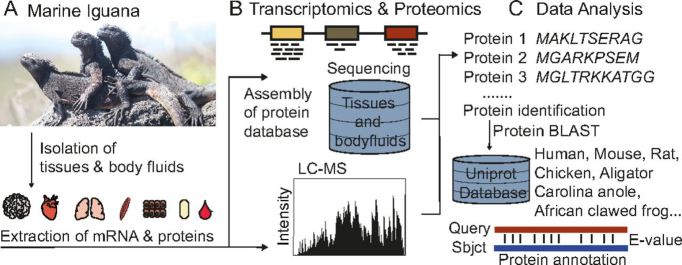
**Deciphering the Galápagos marine iguana proteome by integration of transcriptomics and proteomics.** Workflow from tissue isolation (*A*) to database generation (*B*) and to data analysis (C).

We isolated several tissues, including brain, muscle, heart, and skin, from a freshly dead marine iguana found on Genovesa Island. Moreover, we collected femoral gland secretions, seminal fluid, and blood from living animals on the same island (Fig. 1). First, we performed transcriptomic analysis of five different tissues (brain, heart, lung, muscle, skin), as well as isolated erythrocytes to generate the reference protein database (11). Bioinformatic analysis of the data set revealed 499,046 contigs, mostly ranging in size from 300 – 1000 nucleotides, resulting in an N50 value of 1362 (supplemental Fig. S1A, S1B) (15). We *de novo* assembled the marine iguana transcriptome using all six potential reading frames (32), and the transcript sequences were translated into 105,810 protein open reading frames (ORFs), which served as a database for peptide identification (supplemental Fig. S1A).

**Fig. 2:**
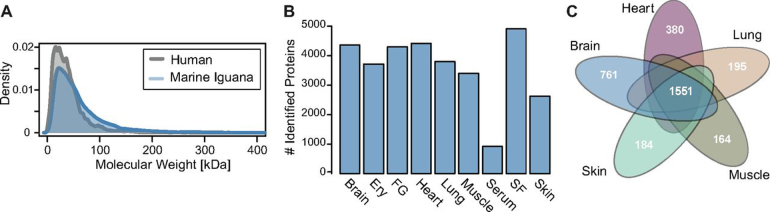
**Metrics of marine iguana proteomics data.***A*, Distribution of protein molecular weight of human proteome and marine iguana proteome. *B*, Number of proteins identified in tissues and secretions; Ery is erythrocytes, FG is femoral glands secretion and SF is seminal fluid. *C*, Overlap of identified proteins between tissues.

Proteins were extracted from tissue samples (Fig. 1*A*) and digested via in-gel digestion. Tryptic peptides were identified using 85 LC–MS/MS measurements, each with a gradient of 150 min. We identified 97,804 different peptides that matched to 7513 protein database entries with false discovery rates (FDRs) below 1% at the peptide and protein levels (Additional file 1). Half of the detected proteins were identified with a sequence coverage of > 23% (supplemental Fig. S1C). On average, we mapped ∼4000 proteins per tissue with a molecular weight distribution resembling the human proteome (Fig. 2*A*) and ∼900 proteins in the serum sample, indicating the high dynamic range for this body fluid (Fig. 2*B*) (33). Next, we overlapped the proteins identified in five solid organs and observed that 1551 proteins were expressed in all analyzed tissues (Fig. 2*C*), and 499 proteins were present in both the fluid samples (serum, seminal fluid, gland secretions) and isolated erythrocytes (supplemental Fig. S1D).

Because our transcriptome data set was based on a *de novo* assembly approach, ORF identification by itself does not provide any gene or protein information. Therefore, we sought to annotate the iguana proteome via BLAST protein sequence similarity searches against several related organisms, including the Carolina anolis (*Anolis caroliniensis*), alligator species (*Alligator mississippiensis*, *Alligator sinensis*), as well as chicken, mice, and human databases. Furthermore, we selected known marine iguana protein entries and those of two closely related iguana species (the green iguana *Iguana iguana* and the Galápagos land iguana *Conolophus subcristatus*) for identification of marine iguana proteins (supplemental Fig. S1E, S1F). Because of the evolutionary distance between the marine iguana and the selected species, we chose a stringent E-value < 10e-20 to ensure detection of highly similar proteins. In total, we transferred 7251 annotations from other species, which represents an annotation efficiency of 96.5% (Additional file 1). To rule out annotation-bias toward highly abundant proteins, we clustered all proteins using their -log10 E-values, based on the sequence alignments against several organisms in the UniProt database. Protein intensities were plotted for each protein (supplemental Fig. S1E, S1F) on the resulting heat map. For example, comparison of muscle tissue from mouse and marine iguana revealed similar intensities for high abundance creatine kinase (CKM), whereas both samples showed low abundance of the E3 ubiquitin-protein ligase (UBR3) (supplemental Fig. S1G), supporting an unbiased protein annotation across protein intensities.

Notably, protein annotation using the protein BLAST search approach led to simultaneous annotation of several proteins to the same UniProt entry (Additional file 1). For example, seven marine iguana proteins were annotated to the human cytosolic phospholipase A2ε (PLA2G4E; supplemental Fig. S2A). Based on this observation, we screened the detected peptides and found unique peptides representing different phospholipase isoforms in the marine iguana data set. We therefore suggest this protein has greater diversity in the marine iguana than in mammalian species.

##### Identification of Proteins with No Orthologs to Other Organisms

To characterize proteins that show no recognizable sequence similarity to other species (Additional file 1), we predicted their subcellular localization using DeepLoc software (22). Thirty-two proteins were predicted to localize in the nucleus, 21 to the mitochondria, and 15 to contain signal peptides that indicate extracellular localization. Among these 15 candidates, two were also enriched in femoral gland secretions (supplemental Fig. S5; Additional file 1). Last, a domain search (24) identified 38 distinct Pfam domains on 28 proteins, of which four are restricted to femoral gland secretions. For example, the protein (TR246911|c0_g3_i1|m.144323) contains a four-disulfide core domain, similar to the antileukoproteinase SLPI. This class of proteins, also described as whey acidic proteins (WAP), has also been detected in crustaceans (34), as well as in mammalian lungs (35). In addition, a single BLAST search with TR246911|c0_g3_i1|m.144323 only revealed an overlap to the nawaprin protein (E-value of 2e-14; 60% identity) expressed in the black-necked spitting cobra (*Naja nigricollis*). This extracellular protein—with potential peptidase inhibitor activity—might damage bacterial cell membranes.

Using these methods, we found no homology for 121 proteins (supplemental Fig. S5). To further shed light on the identity of these proteins, we performed a BLAST search using an extended set of species, including the Coral snakes *Micrurus spixii* and *M. surinamensis*. To identify also distant proteins we used the BLAST search without any E-value restriction. The resulting list of potential homologs contained proteins with E-values ranging from 0.31 to 2.5E-157. We grouped these E-values to different confidence classes, with 25 high confidence homologs, 48 low confidence homologs and 49 proteins without homologs (E-value >1E-05) (supplemental Fig. S5). We speculate that the latter 49 proteins may represent proteins specific for the Iguanidae. However, a more detailed comparison with proteomic data from other related lizard species is required to verify whether these proteins are also present in other members of the Iguanidae or if they are specific to the marine iguana.

##### Proteome of the Marine Iguana Femoral Gland Secretions Contains Epidermal Marker and Immunregulatory Proteins

**Fig. 3:**
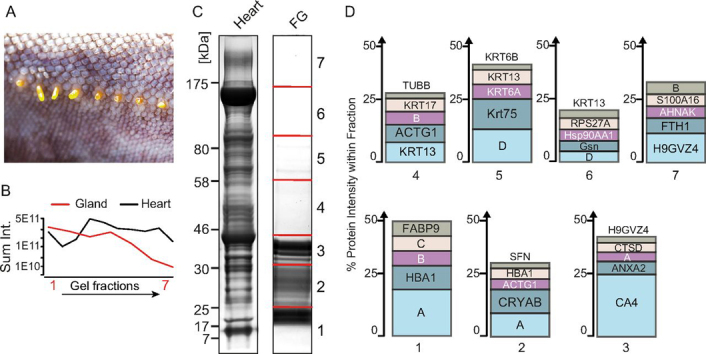
**Identification of proteins enriched in the marine iguana femoral gland.***A*, The femoral glands with secretions. *B*, Sum intensity of proteins per gel fraction in marine iguana femoral gland secretions and heart. *C*, Coomassie-stained protein gel of femoral gland secretions from heart and marine iguana. Molecular weight indicated on the left in kDa, Fraction indicated on the right. FG is femoral gland secretion. *D*, Most abundant proteins in each fraction of the femoral gland secretions. *A*: TR63421|c0_g1_i1|m.7934; *B*: WAP four-disulfide core domain protein 3-like (from the Chinese Alligator); C: Beta-microseminoprotein (from the American Alligator) *D*: TR238240|c0_g1_i1|m.134007.

To analyze the protein compounds, femoral gland secretions were isolated by carefully squeezing the femoral glands of the marine iguana (Fig. 3*A*). The emerging substance was immediately homogenized in a denaturing buffer and separated by SDS-PAGE gel electrophoresis. A Coomassie staining of separated proteins revealed lower protein mass distribution in marine iguanas (ranging from ∼15–40 kDa), when compared with protein extracts from the heart of the same animal (Fig. 3*C*).

We characterized the marine iguana femoral gland secretions by protein BLAST searches, protein abundance, and similarity to other tissues to identify proteins that are femoral gland specific or enriched in femoral gland secretions. MS analysis identified 4305 proteins in femoral gland secretions of which 4111 proteins could be identified with at least two peptides (see Additional file 1). Consistent with the Coomassie protein staining results, the total protein intensity and number of proteins identified per gel fraction substantially declined by one order of magnitude from band 1, containing the lowest molecular weight proteins to band 7 with the highest molecular weight (Fig. 3*B*).

**Fig. 4:**
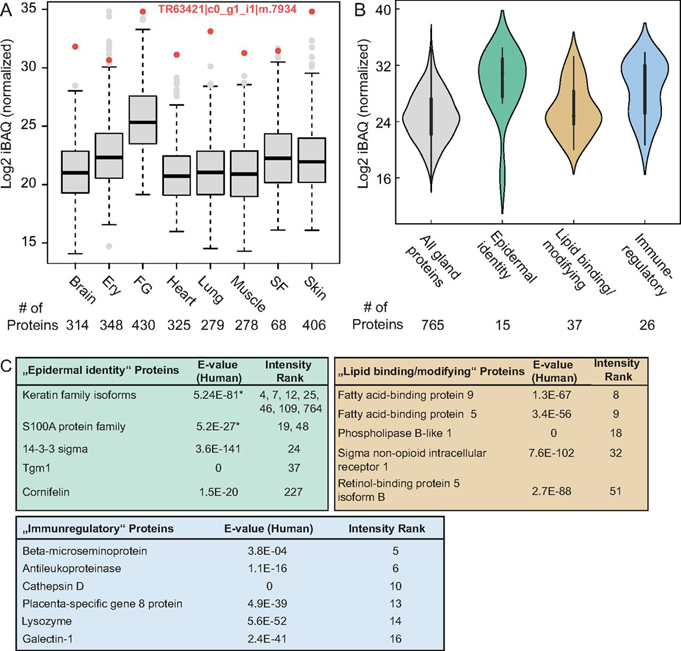
**Expression of immunoregulatory proteins in femoral gland secretions.***A*, iBAQ intensity of the proteins enriched in femoral gland secretions in solid tissues and body fluid samples. FG femoral gland secretion and SF is seminal fluid. *B*, Intensity distribution of proteins enriched in femoral gland secretions. *C*, Selected secretion-specific proteins. *Depicted E-value corresponds to the protein with the lowest rank number.

We determined the five most abundant proteins in each marine iguana femoral gland secretion gel fraction. In the first fraction, the most abundant protein (A) was a 12 kDa protein, which represented ∼20% of the total intensity (TR63421|c0_g1_i1|m.7934) (Fig. 3*D*). This protein was identified by 16 unique peptides and is detectable in all tissues analyzed, with the highest abundance in the femoral gland (Fig. 4*A*). Moreover, sequence alignments to other organisms at DNA and protein levels did not reveal any ortholog candidate or conserved protein domain. This protein may indicate the product of a fast evolving orphan gene, which lost sequence similarity within a short evolutionary timespan (36, 37). Furthermore, we found carbonic anhydrase 4 (CA4) as the most abundant protein with about 25% in fraction 3; this protein likely functions to regulate the pH of secretions by balancing the bicarbonate level. The detection of several keratins in the fractions 4-6 and 14-3-3 sigma (also known as epithelial cell marker protein 1) reflect the epidermal origin of femoral glands.

Femoral glands are holocrine glands, thus their secretion is composed of whole cells and includes cytosolic, cytoplasmic and secreted proteins. Metabolic and house keeping proteins in femoral gland secretions are likely shared with other cell types and tissues, which prompted us to decipher femoral glands specific proteins. To define a gland-specific proteome, we overlapped all detected femoral gland proteins with solid tissue and body fluid proteins, and found 337 proteins that are unique to femoral gland secretions (Additional file 1). To complement the set of unique femoral gland (FG) proteins with enriched FG proteins, we performed k-means clustering and identified a cluster of proteins enriched in FG, and to a lesser extent, in the seminal fluid (SF) (supplemental Fig. S3A–S3B). To further specify femoral gland enriched proteins, we applied a principal component analysis (PCA) of z-score transformed iBAQ protein intensities for SF and FG (supplemental Fig. S3C). We used PCA loadings and k-means cluster information to define a subset of 428 proteins (supplemental Fig. S3D, green circles) that are enriched in femoral gland secretions (Additional file 1).

Ranking these femoral gland-specific proteins (337 + 428 = 765) by their protein intensity enables the identification of several epidermis specific proteins, including epiplakin (EPPK1) and cornifelin (Fig. 4*B*, Additional file 1). In addition, we identified a large number of proteins with lipid-binding and lipid-modifying properties, which is consistent with the hydrophobic nature of these secretions.

Interestingly, the FG specific proteome contained proteins with human homologs that have immune responsive or host defensive properties, such as Lysozyme C, Galectin-1, and Antileukoproteinase (Fig. 4*B*–4*C*). To investigate this further, we annotated human homologs with Gene Ontology (GO) terms and found 68 proteins annotated with “activation of innate immune response,” and 32 proteins with “adaptive immune response” (GO: 0002250). We manually inspected the proteins with highest expression values in femoral glands and found that some proteins harbor antimicrobial properties, including Protein S100-A7, beta-microseminoprotein (MSMB, PSP94) (38), and the complement system components C5-9. In addition, three proteins were annotated as potential orthologs of mouse C-type lectin pulmonary surfactant-associated proteins, which are important components of the innate immune system as a direct defense against pathogens, and are present in the fluid lining the surface of lung alveoli. Pulmonary surfactant-associated protein D (SFTPD) belongs to the collectin protein family, and contains an N-terminal collagen-like domain and C-terminal lectin domain (39).

##### Peptides from Femoral Glands with Antimicrobial Activity

**Fig. 5:**
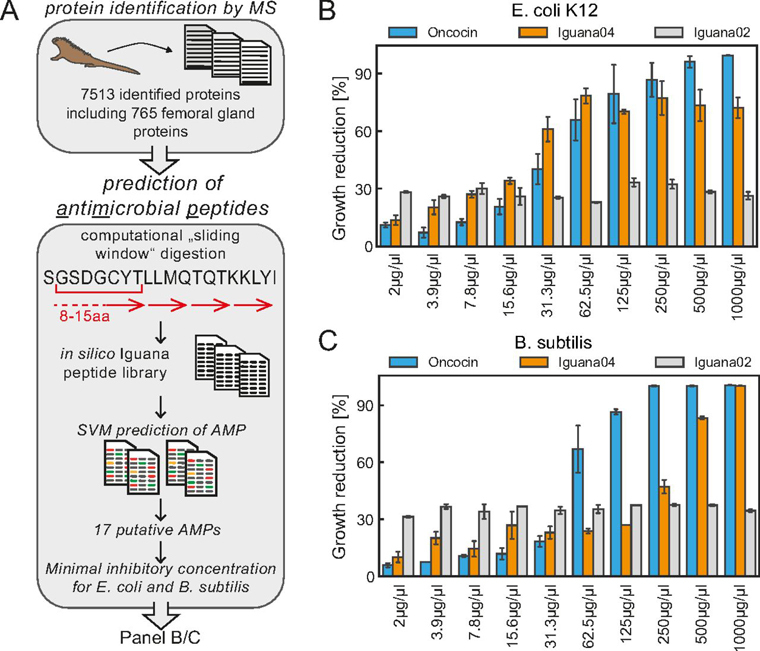
**Screen for anti-microbial peptides in marine iguana proteome.***A*, Working scheme for prediction of anti-microbial peptides (AMPs). *B*, Minimal inhibitory concentration of marine iguana peptides “Iguana-04” and “Iguana-02” in Escherichia coli K12 cultures. *C*, Minimal inhibitory concentration of marine iguana peptides “Iguana-04” and “Iguana-02” in Bacillus subtilis cultures. The known AMP oncocin served as a positive control for bacterial growth reduction in figures (*B*) and (*C*).

We were intrigued by these antimicrobial signatures and sought to systematically analyze the proteome of femoral glands. To this end, we utilized the marine iguana protein database of all detected proteins, and generated a library of potential antimicrobial peptides (AMP) by *in-silico* sliding window digestion. Here, we generated sequence windows of 8 to 15 amino acids and resulting peptides were predicted for antimicrobial activity by a trained support vector machine (as described in the methods) (Fig. 5). This step yielded in 8675 potential candidates and we selected a subset of 17 AMPs from 13 proteins with high water solubility and stability to avoid problems in solid phase peptide synthesis (Additional file 2). Of the selected peptides with predicted antimicrobial activity, nine were derived from proteins that showed an accumulation in the femoral glands and four were from proteins evenly expressed in all tissues analyzed. To experimentally test the antimicrobial properties of these peptides, we determined growth rate reductions of bacterial cultures in the presence of increasing candidate peptide concentration. As positive control, we used the well-established AMP oncocin (40). As negative control, we have picked four peptides predicted to have no AMP activity. The analysis showed a moderate inhibitory activity for 14 peptides, partly without dose dependence (Additional file 2). However, peptide #4: KKLYISKGCMSKSLC demonstrated a strong antimicrobial effect against *E.coli* and *B. subtilis* cultures with a dose dependent maximal growth reduction of approx. 75% in *E. coli* cultures and 95% in *B. subtilis* cultures. Although *B. subtilis* cultures require high concentrations of “Iguana #4” for maximal growth reduction, this peptide exceeded even the oncocin activity in *E. coli* cultures at low concentrations between 3 and 60 µg/µl. The selected negative controls did not show any antimicrobial activity (Additional file 2).

## DISCUSSION

The ancestral lineage of Galápagos iguanas diverged around 5–6 million years ago into the land and marine iguana lineages. Unique among lizard species worldwide, marine iguanas have evolved several distinctive adaptations to cope with the conditions of the marine environment. To date, there has been no systematic attempt to unravel the functions underlying these adaptations through transcript or protein analysis. Here, we report the first comprehensive *de novo* transcriptome assembly, together with a protein atlas for several tissues and body fluids of this sea-going iguana. In total, we identified over 100,000 transcripts and 7513 experimentally verified protein sequences.

Among the different organs, the femoral gland plays a central role in chemical communication and territorial demarcation in lizards and amphisbaenians. Although earlier studies identified a plethora of secreted lipophilic and volatile substances, work to identify the proteins that constitute the major fraction of femoral gland secretions is entirely lacking. Proteins have been assumed to play also a major role in chemical communication (7, 9), similar to the function of secreted volatiles, which serve as olfactory pheromones. Because substrate licking is present in several members of the Iguanidae family, marine iguanas can possibly detect proteins via their vomeronasal organ to recognize occupied territories and to find potential mating partners. Perception of nonvolatile substances, such as proteins and peptides via olfactory receptor neurons or the vomeronasal organ for attraction and recognition of individuals are well described for mammals. The major urinary proteins (MUPs), for example, provide information to other individuals when recognized via olfactory sensing. Secreted MUPs function as pheromone transporters, stabilize volatile substances, provoke aggression, and play a role in attraction of mating partners (41). At a first glance, the analysis of more than 4000 femoral gland-secreted proteins indicated no functional correlates with MUPs or other proteins involved with communication, therefore, communication may occur through currently unknown secreted proteins.

Mass spectrometry showed a prevalence of femoral gland-secreted proteins within a low molecular mass range (below ∼40 kDa). Degradation can be excluded, because the protein migration patterns correlated with their predicted masses (supplemental Fig. S2D). However, conclusive evidence explaining why smaller proteins are overrepresented in femoral gland secretions is lacking and this topic should be addressed in future studies. Also, the comparison of femoral gland proteins to other squamate species may help to unravel whether such low molecular weights are restricted to marine iguana secretions or is a general pattern across squamates.

The high number of cellular proteins reflects the presence of keratinocytes, granular cells, and epithelial cells in the glandular parenchyma as holocrine organs (42). Not surprisingly, keratin isoforms comprised ∼10% of the secreted proteome and together with cholesterol are the major components of femoral gland matrix. Further, we found two fatty acid binding proteins (FABP5, FABP9) with high signal intensities. FABP5 is expressed in the epidermis and transports fatty acids and other lipophilic substances, such as eicosanoids. FABPs also function as lipid chaperones and are involved in the conversion of fatty acids to eicosanoid intermediates, which are present at high levels in marine iguana femoral gland secretions signaling male quality (9).

##### Femoral Gland Secretions as a Barrier to Microbial Infection and a Preservative Matrix

We identified in the femoral gland secretions a highly abundant protein with a high level of homology to human lysozyme C. This immune-related protein with a bacteriolytic function provides the first hint that femoral gland secretions may also function as a barrier to microbial infection. Earlier studies showed that secreted galectins (*e.g.* LGALS1) modulate cell-matrix adhesion (43). LGALS1 also functions as an immunosuppressor for T cells, and has recently been shown to be expressed at high levels in secretory granules released from cytotoxic T lymphocytes (44). Detection of placenta-specific gene 8 (PLAC8) and three orthologs of SFTPD (supplemental Fig. S4) further indicates the presence of components of the innate immune system. Thus, we speculate that marine iguana femoral glands are equipped with a battery of immune cells that protect marine iguanas against infections through the openings of glands.

As a side effect, anti-microbial proteins may also prevent the rapid degradation of femoral gland secretions by bacteria. The fatty secretions produced by the femoral glands, which are located on the ventral hind limbs of males, are actively or passively smeared onto the ground or rocks to mark a males' territory. Such volatile substances could be rapidly degraded by bacteria under the hot and humid conditions of the marine iguana's terrestrial environment, therefore an anti-microbial mixture of proteins and peptides could conserve the secretions and thus sustain the signaling function for a longer duration.

Although our sequence alignment efforts identified direct orthologs in other species for more than 98% of the proteins, several proteins did not show any correlation with existing databases, indicating the existence of marine iguana-specific proteins. For example, TR63421|c0_g1_i1|m.7934 has no homology with other species, no conserved sequence motifs, and is expressed at the highest level in the femoral gland. We would like to emphasize that other members of the Iguanidae family could express similar proteins. The generation of transcriptome databases for the Galápagos land iguana and lizards of the genus Ctenosaura—the phylogenetic sister group of Galápagos iguanas from mainland South America—could clarify whether these proteins have evolved in the Galápagos lineage or only in marine iguanas. Further studies are also needed to explore whether these proteins play a specific role in chemical communication or serve other biological function.

In contrast to the overall expectation that proteins secreted by femoral glands of lizards should function in chemical communication, we found instead clear evidence for a complex and rich molecular barrier to microbes and other potential pathogens. This work adds to previous studies that have identified protein fragments with antimicrobial activity (45, 46, 47). Based on a comprehensive catalog of femoral gland-secreted proteins, this study opens up a new avenue of research addressing the functions and potential applications of such proteins. The detection of several novel protease inhibitors and C-type lectin proteins may provide a basis for identification of new therapeutic proteins and inhibitors for protease-induced inflammatory responses in human lung- and skin diseases.

In addition, our proteomics study showed that the femoral glands might be a source for novel biologically active AMPs. A more focused analysis of processed peptides from the femoral secretions by the use of specific protocols, including removal of proteins, deactivation of enzymes, and specific peptide separation techniques such as capillary electrophoresis and top-down fragmentation (48) should be utilized to identify more AMPs. Because many AMPs are only expressed and processed after a bacterial infection (49, 50), a screening of the gland secretions of animals with varying health statuses could be another option to identify candidates and thus lead toward the development of potential new antibiotics.

## CONCLUSIONS

By the use of proteomics we analyzed proteins in femoral gland secretions of the Galápagos marine iguana. In this species, femoral gland secretions contribute to male quality signaling and mate recognition. Although we found no evidence for proteins and peptides involved in chemical communication, we identified several immune-regulatory proteins, which demonstrate anti-microbial functions. Accordingly, we show that femoral gland proteins and peptides function as a barrier against microbial infection and may prevent the rapid degradation of volatile substances.

## DATA AVAILABILITY

The MS proteomics data have been deposited to the ProteomeXchange Consortium via the PRIDE partner repository (51) with the data set identifier PXD018909.

The RNAseq assembled data are available at NCBI BioProject repository, identifier PRJNA602224. link: https://dataview.ncbi.nlm.nih.gov/object/PRJNA602224?reviewer=n4lrjmp7gvclbnhr84rt70okpt
