# Supplementary material for: Proteomics of Galápagos Marine Iguanas Links Function of Femoral Gland Proteins to the Immune System
Source: Mol Cell Proteomics. 2020 Nov 25;19(9):1523–32. doi: 10.1074/mcp.RA120.001947 (PMC8143647; doi:10.1074/mcp.RA120.001947)
Supplement: Supplementary file 1 [file mmc1.zip › mmc1/158307_1_supp_525666_q9x0f1.docx]

The datasets supporting the conclusions of this article are included within the article and its additional files:

- Additional file 1.xlsx, proteomics results table including BLAST results. This file contains comprehensive results about all detected proteins in various organs of marine iguanas as well as BLAST results for each protein. Further, this file contains computational predictions for a selection of proteins for localization and domain homology.
- Additional file 2.xlsx, parameter for SVM based AMP predictions and AMP validation.
- Additional file 3.pdf, additional information about materials, methods and results. This file includes extended information about materials and methods and provides five additional figures, Fig. S1-Fig. S5.
