# Supplementary material for: Proteomics of Galápagos Marine Iguanas Links Function of Femoral Gland Proteins to the Immune System
Source: Mol Cell Proteomics. 2020 Nov 25;19(9):1523–32. doi: 10.1074/mcp.RA120.001947 (PMC8143647; doi:10.1074/mcp.RA120.001947)
Supplement: Supplementary file 1 [file mmc1.zip › mmc1/158307_1_supp_528142_qf67yk.pdf]

Supporting Information

SI Material and Methods

RNA isolation and transcriptomics

Isolated iguana tissues were stored in RNAlater to stabilize and preserve RNA. RNA was isolated from tissues using the miRNeasy micro kit (Qiagen) combined with on-column DNase digestion (DNase-Free DNase Set, Qiagen) to avoid genomic DNA contamination. RNA and library preparation integrity were verified using a BioAnalyzer 2100 (Agilent) or LabChip Gx Touch 24 (Perkin Elmer). Total RNA (1–4 µg) was used as input for TruSeq Stranded mRNA Library preparation, following the low sample protocol (Illumina).

Protein isolation and digestion

Tissues and body fluids were lysed in SDS buffer (4% SDS in 100 mM Tris/HCl, pH 7.6). Lysates were homogenized, heated at 70 °C for 10 min, clarified by centrifugation and protein concentrations were determined using the Bio-Rad DC assay. Proteins (40 µg) were separated using precast 4–12% Bis-Tris gels (Invitrogen) and in-gel digestion was performed {Shevchenko, 2006 #29}. Briefly, after Coomassie staining, each lane was cut into seven slices, followed by de-staining of the gel pieces, reduction with 10 mM dithiothreitol (DTT) at 56 °C for 45 min and carbamidomethylation with 55 mM iodoacetamide (IAA) in the dark for 30 min at room temperature. Proteins were digested using the proteases LysC (Wako) and Trypsin (Promega) overnight at 37 °C, peptides were extracted in acetonitrile and, after removal of organic solvent using a SpeedVac concentrator, the samples were acidified with trifluoracetic acid. Desalting was performed using Stop and Go extraction tips {Rappsilber, 2007 #30}.

For in-solution digestion, samples were dissolved in 8 M urea buffer (6 M urea, 2 M thiourea in 10 mM HEPES, pH 7.6) {Nolte, 2014 #463}. Samples were reduced with 10 mM dithiothreitol (DTT) for 30 min, followed by carbamidomethylation with 55 mM iodacetamide

(IAA) in the dark for 30 min at room temperature. Proteins were digested with LysC for 2 h, diluted to 2M with urea, then digested overnight with trypsin. The reaction digestion was stopped by adding an equal amount of Buffer C (5% CAN, 1% trifluoroacetic acid) and the peptides were desalted using in-house made C18-based Stop and Go Extraction Tips.

LC-MS/MS analysis

Mass spectrometric analysis was performed using an Easy nLC 1000 UHPLC coupled to a QExactive mass spectrometer (Thermo Fisher), as previously described {Nolte, 2014 #463}. Peptides were fractionated using self-made made 50 cm columns packed with 1.9 µm C18 beads using a binary buffer system, consisting of Buffer A (0.1% FA) and Buffer B (80% ACN in 0.1% FA). Peptides were eluted from the C18 column by applying a linear gradient from 5–35% buffer B over 150 min.

MS data processing

The raw data acquired were analyzed using MaxQuant software (1.5.3.8) {Cox, 2011 #14} using the endoprotease trypsin as digestion enzyme and allowing a maximum of two missed cleavages. Oxidation of methionine and *N*-terminal acetylation were selected as variable modifications and carbamidomythylation of cysteine as a fixed modification. The maximum mass tolerance was set to 4.5 ppm in the main search, initial precursor ion mass deviation to 7 ppm, and MS/MS tolerance to 20 ppm.

Data analysis

To identify proteins enriched in femoral gland secretions in an unbiased manner, we employed a strategy combining kmeans clustering and principal component analysis (PCA). First, NAs were imputed using the *R* package mice {van Buuren, 2011 #407}. To account for technical variations while comparing different tissues and body fluids, quantile normalization was applied to the iBAQ intensities of each sample using the *R* package limna {Ritchie, 2015 #408}. Normalized intensities were imported into Instant Clue and transformed into *z*-scores. *Z*-score

profiles were separated into eight clusters by kmeans clustering; the cluster with high *z*-scores in femoral gland secretions but low scores in other tissues was chosen. Since the center profile of this cluster exhibited a higher value in seminal fluids, PCA was used for further separation.

SI Figure Legends

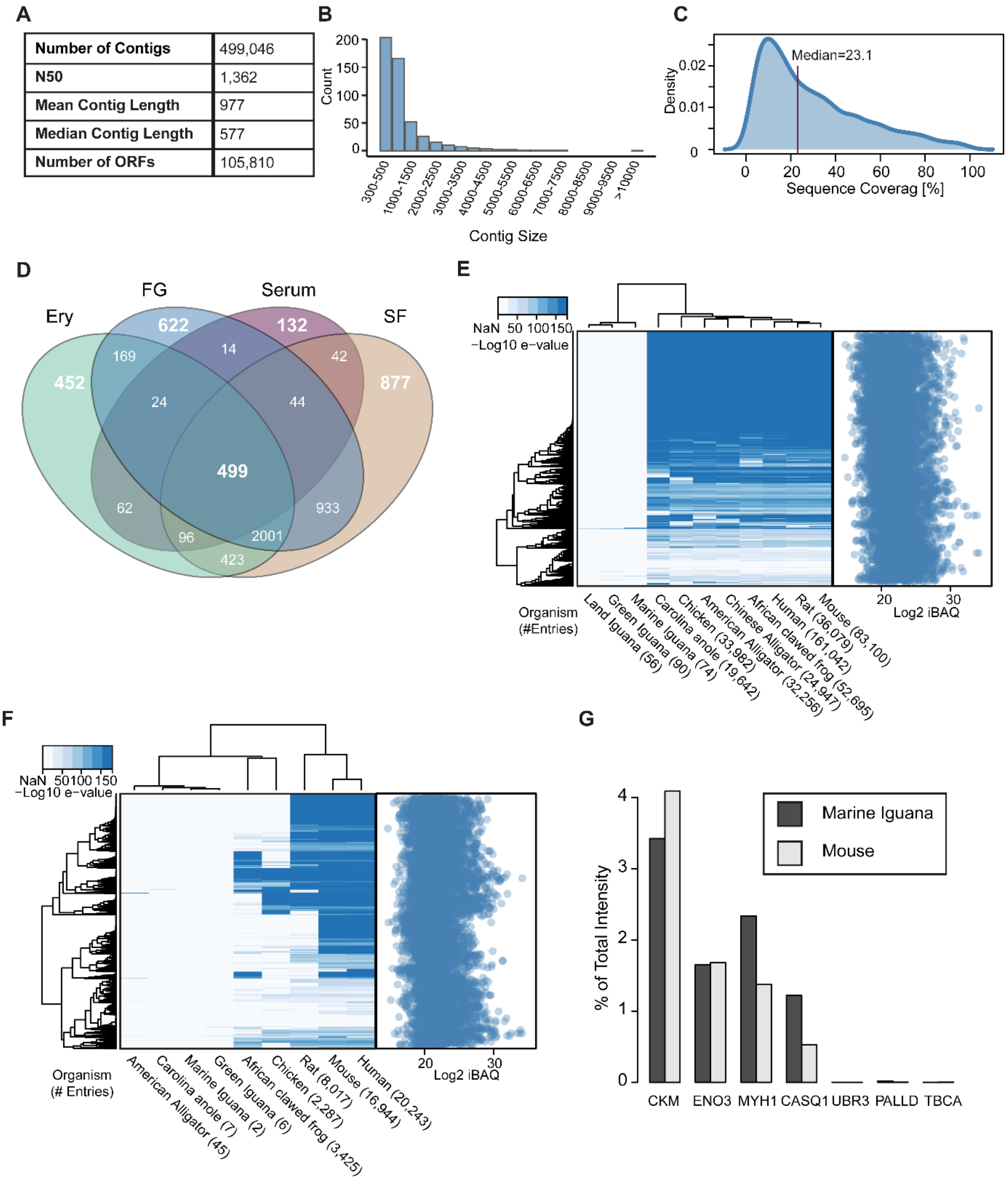

Fig. S1: (A) Statistics of transcript sequencing. Distribution of (**B**) contig size and (**C**) protein sequence coverage. (**D**) Overlap of identified proteins in fluids/secretions. Ery is erythrocytes, FG is femoral gland secretions and SF is seminal fluid. Heatmaps of –log10 E-value resulting from BLAST searches against (**E**) the whole UniProt database and (**F**) a restricted search against the SwissProt database. Protein intensities were plotted for the clustered proteins. (**G**) Relative abundance of selected proteins in marine iguana muscle and a mouse muscle proteomics data set.

A

MARINE IGUANA	Molecular Weight [kDa] MS-identified Protein	BLAST Hit HUMAN Swissprot/Uniprot	Molecular Weight [kDa] BLAST Hit	Number of Unique Peptides
TR165827|c1_g1_i2|m.39637	58			3
TR165827|c2_g2_i7|m.39645	97	Q3MJ16-3	98	35
TR174861|c0_g1_i1|m.50504	98			15
TR194012|c1_g1_i1|m.76559	72			38
TR202985|c0_g1_i2|m.87583	21	Q3MJ16-2	57	6
TR205630|c0_g1_i6|m.90883	95	Q3MJ16-3	98	44
TR220600|c8_g2_i1|m.112106	94	Q3MJ16-3	98	13

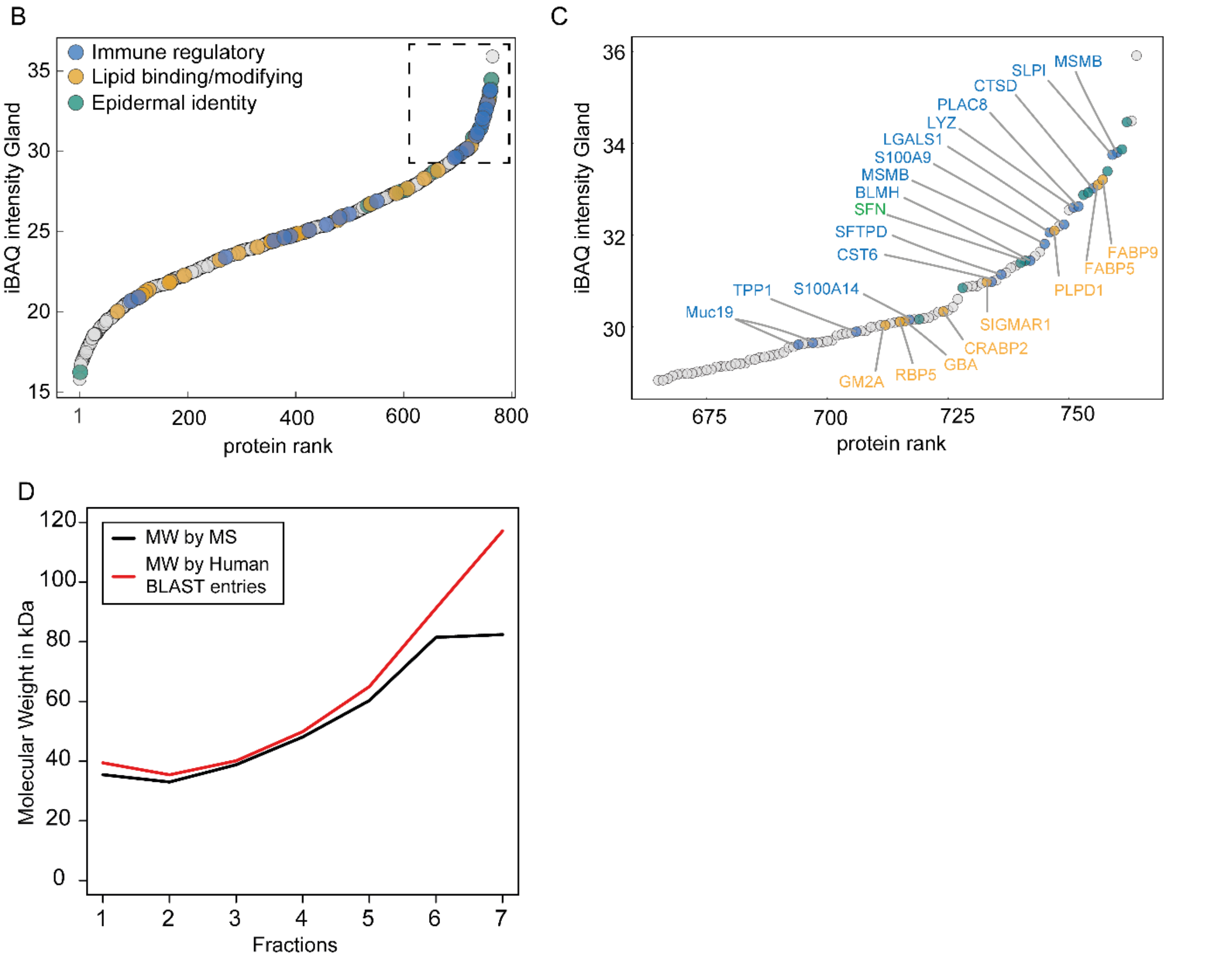

Fig. S2: (**A**) Marine iguana proteins annotated with a human UniProt entry and the associated numbers of unique peptides. (**B**) Ranked iBAQ intensity of all proteins specific for or enriched in femoral gland secretions. Inset shown in C. Blue: Proteins with antimicrobial properties, orange: proteins with lipid binding or modifying properties, green: proteins with epidermal identity (keratins) and 144-3-3 sigma (SFN) **(C)** Inset of B. Keratins and 14-3-3 sigma (SFN)

in green. (**D**) Mean molecular weight of femoral gland secretion gel fractions and corresponding molecular weight of human BLAST hits.

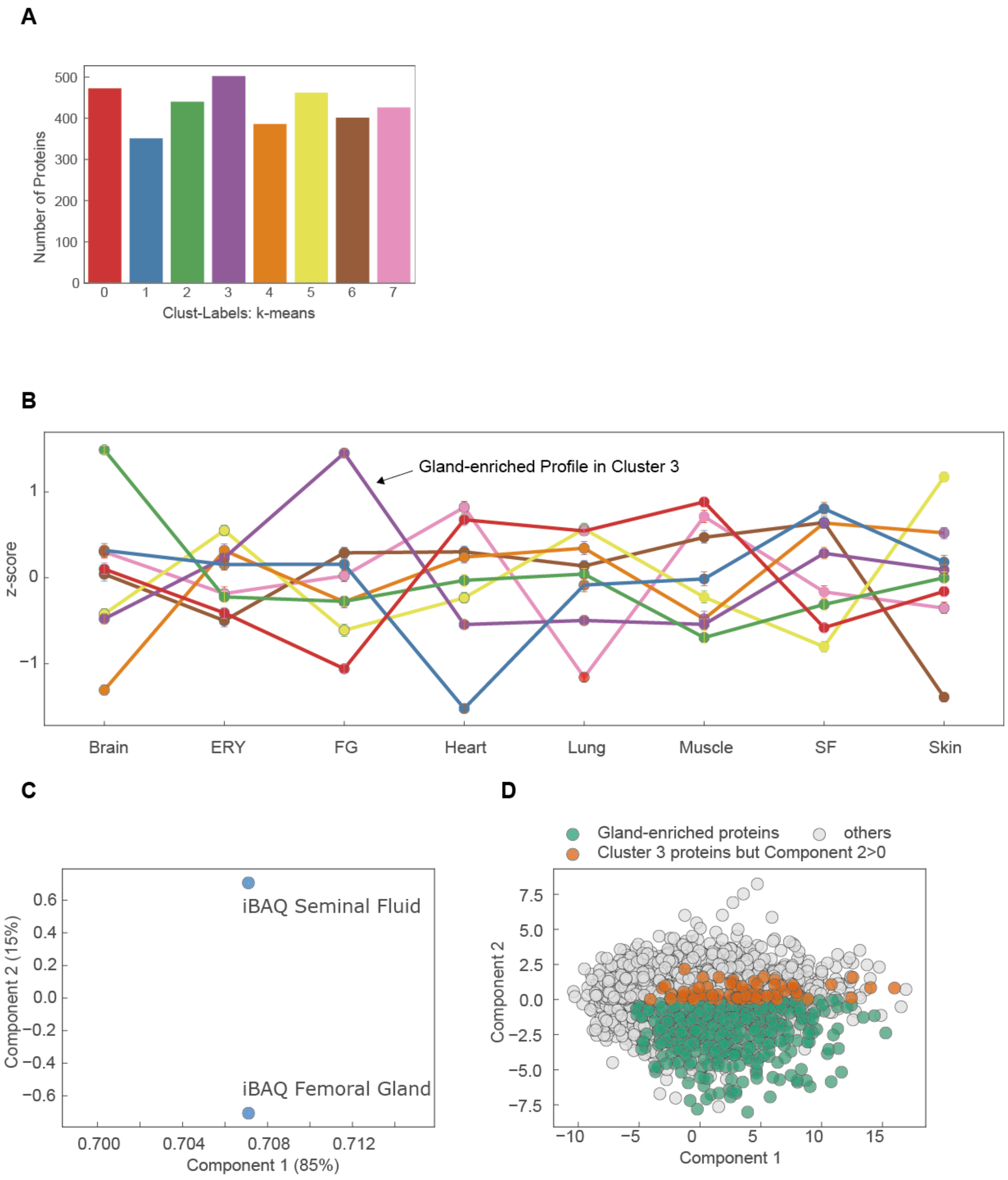

Fig. S3: Identification of proteins enriched in femoral gland secretions. **(A)** Number of proteins per cluster resulting from kmeans clustering. **(B)** *Z*-score profiles of each cluster across all tissues and body fluids. Cluster three exhibited high z-scores in femoral gland secretions. **(C)** Projections of PCA of proteins in femoral gland secretions and seminal fluid. **(D)** Loadings for

PCA. Proteins indicated by green dots with component 2 < 0 represent proteins enriched in femoral gland secretions.

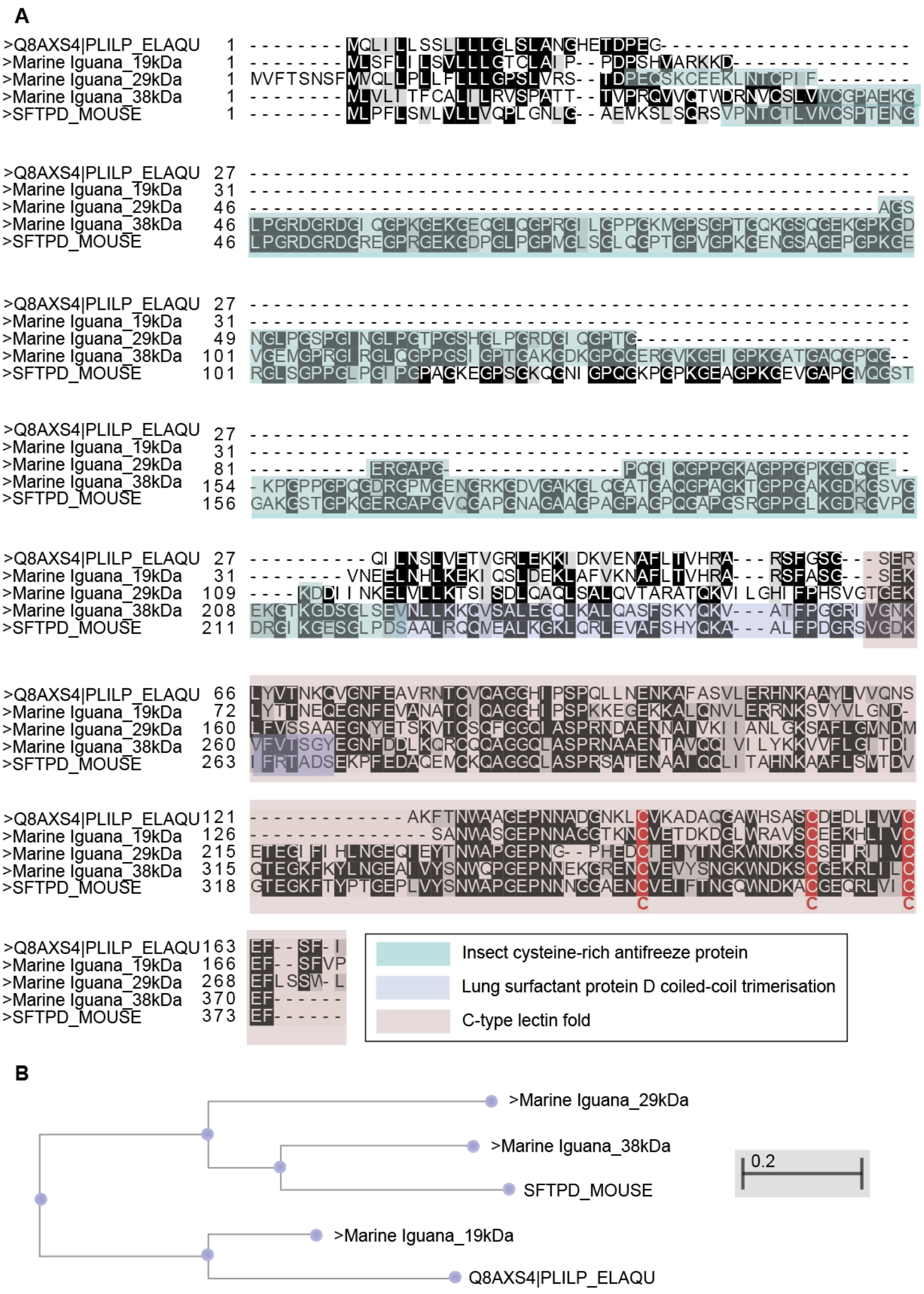

Fig. S4: **(A)** Multiple sequence alignment of mouse Surfactant Protein D (SFTPD), Japanese four-lined ratsnake PLIα-LP and marine iguana proteins annotated with SFTPD protein domains via sequence analysis with INTERPRO. (**B**) Phylogenetic tree based on the multiple

sequence alignment in (A) generated using COBALT. In femoral gland secretions, we identified a shorter variant that overlaps with the C-terminal C-type lectin domain of mouse SFTPD, but lacks the collagen and antifreeze domain.

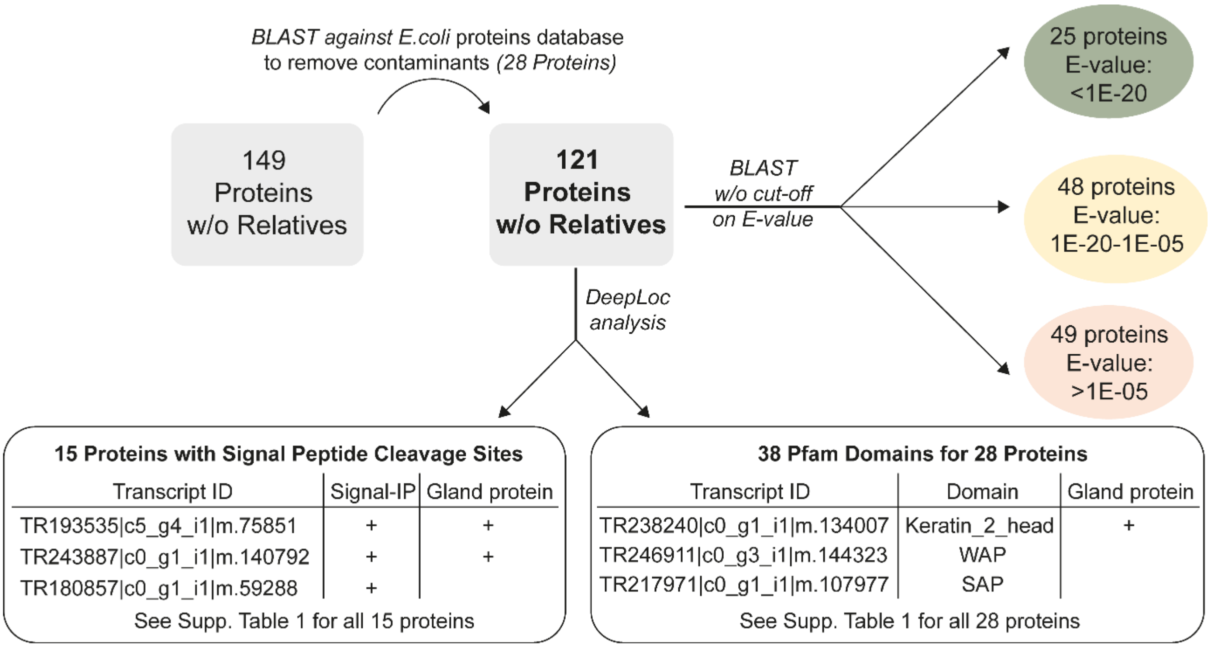

Fig. S5: Further characterization of not annotated proteins using BLAST search against UniProt and SwissProt protein sequences and bioinformatic tools, including protein domain searches. Proteins with similarities to *Escherichia coli* proteins were removed.
